# “Weight loss, Semaglutide and Manic Episode”: A case report

**DOI:** 10.1192/j.eurpsy.2024.452

**Published:** 2024-08-27

**Authors:** J. Romay Gonzalez, P. Hernández Liebo, C. Sevilla Diez, O. Anabitarte Bautista, L. Cayon de la Hoz, R. Obeso Menendez, M. Polo Gay, M. Hoyuelos Cobo, G. Cortez Astudillo

**Affiliations:** ^1^Psychiatry, Hospital Universitario Marqués de Valdecilla, Santander, Spain

## Abstract

**Introduction:**

The glucagon-like peptide-1 (GLP-1) receptor agonist Semaglutide has been widely used to manage type 2 diabetes due to its favourable effects on glycemic control and weight reduction. Proved to be safe in adults and elderly patients with renal or hepatic disorders demanding no dose modification. Affective symptoms are not listed as side effects in the product information. However, there is a recent investigation going on by the European Medicines Agency (EMA) after three flagged cases of suicidal thoughts in Iceland. In contrast, the Food and Drug Administration (FDA) recommend that patients with this treatment are monitored for suicidal thoughts or behaviour.

**Objectives:**

This case study explores the possible relationship between Semaglutide treatment and the onset of a manic episode in a 57-year-old male with no history of psychiatric disorders.

**Methods:**

We present a 57-year-old male with no psychiatric history of interests, with a previous good functioning. A one-week history of disruptive behaviours started, characterized by excessive cheerfulness, heightened euphoria, and reduced need for sleep. Family members describe a complex situation at home, with frequent outings by the patient, engaging in conversations with strangers, getting lost, and becoming more irritable with them. The patient and family relate this mood change after initiating Semaglutide for diabetes control, starting at 7mg doses. The temporal relationship between the initiation of Semaglutide therapy, precisely a dose escalation to 7mg, and the onset of manic symptoms prompted family members to notify the patient’s endocrinologist. Due to the inability to manage the patient at home and his unpredictability, they sought help at the emergency department, resulting in a psychiatric admission. Imaging and analytical tests show no significant abnormalities.

**Results:**

During his stay in the psychiatry department, semaglutide dosage was reduced, and treatment with Aripiprazole was initiated at doses of 5mg, given the metabolic profile associated with medical comorbidities (obesity, chronic renal failure and diabetes). Subsequent clinical observations showed a gradual resolution of manic symptoms and an improvement in the patient’s overall mental state.

**Conclusions:**

This case highlights the importance of monitoring and recognizing potential neuropsychiatric side effects associated with Semaglutide therapy, particularly in individuals without a prior psychiatric history. Further research is warranted to elucidate the underlying mechanisms linking Semaglutide with mood disturbances and to identify risk factors that may predispose certain patients to develop manic states in response to this GLP-1RA. Clinicians should remain vigilant and consider alternative treatment options if such side effects occur, ensuring comprehensive management of patients receiving Semaglutide for diabetes control.

**Disclosure of Interest:**

None Declared

